# Diurnal cortisol rhythms among Latino immigrants in Oregon, USA

**DOI:** 10.1186/1880-6805-31-19

**Published:** 2012-06-25

**Authors:** Erica C Squires, Heather H McClure, Charles R Martinez, J Mark Eddy, Roberto A Jiménez, Laura E Isiordia, J Josh Snodgrass

**Affiliations:** 1 Department of Anthropology, University of Oregon, 1321 Kincaid Street, Eugene, OR, 97403, USA; 2 Latino Research Team, Oregon Social Learning Center, Eugene, OR, USA; 3 Partners for Our Children, University of Washington, Seattle, WA, USA; 4 Farmworker Housing Development Corporation, Woodburn, OR, USA; 5 Institute of Cognitive and Decision Sciences, University of Oregon, Eugene, OR, USA

**Keywords:** Biomarker, Psychosocial stress, Cortisol, Acculturation, Latino, Immigration, Body composition

## Abstract

One of the most commonly used stress biomarkers is cortisol, a glucocorticoid hormone released by the adrenal glands that is central to the physiological stress response. Free cortisol can be measured in saliva and has been the biomarker of choice in stress studies measuring the function of the hypothalamic-pituitary-adrenal axis. Chronic psychosocial stress can lead to dysregulation of hypothalamic-pituitary-adrenal axis function and results in an abnormal diurnal cortisol profile. Little is known about objectively measured stress and health in Latino populations in the United States, yet this is likely an important factor in understanding health disparities that exist between Latinos and whites. The present study was designed to measure cortisol profiles among Latino immigrant farmworkers in Oregon (USA), and to compare quantitative and qualitative measures of stress in this population. Our results indicate that there were no sex differences in average cortisol AUCg (area under the curve with respect to the ground) over two days (AvgAUCg; males = 1.38, females = 1.60; *P* = 0.415). AUCg1 (Day 1 AUCg) and AvgAUCg were significantly negatively associated with age in men (*P*<0.05). AUCg1 was negatively associated with weight (*P*<0.05), waist circumference (*P*<0.01) and waist-to-stature ratio (*P*<0.05) in women, which is opposite of the expected relationship between cortisol and waist-to-stature ratio, possibly indicating hypothalamic-pituitary-adrenal axis dysregulation. Among men, more time in the United States and immigration to the United States at older ages predicted greater AvgAUCg. Among women, higher lifestyle incongruity was significantly related to greater AvgAUCg. Although preliminary, these results suggest that chronic psychosocial stress plays an important role in health risk in this population.

## Introduction

Chronic psychosocial stress has been linked to negative health outcomes, such as elevated blood pressure, increased susceptibility to infectious disease and a higher risk of developing cardiovascular disease [1]. Psychosocial stressors that have been linked to disease include socioeconomic status (SES), discrimination and lifestyle incongruity (that is, the disparity between an individual’s consumption-based lifestyle and their education or occupational status) [2-4]. Acute psychosocial stress has been shown to activate several physiological pathways, most importantly the sympathetic nervous system (SNS) and the hypothalamic-pituitary-adrenal (HPA) axis; activation of these pathways has the effect of increasing alertness, mobilizing the body’s energy resources and suppressing non-essential functions, such as those associated with growth and reproduction [5,6]. Despite the adaptive benefits in the short-term, repeated activation may lead to the development of health problems because elevated SNS and HPA activity can lead to over-utilization of some functions and suppression of others. Recent evidence also suggests that physiological responses to psychosocial stress may differ depending on the specific stressor (for example, discrimination stress, job stress) [7], but this topic has not been thoroughly investigated.

One of the most commonly used stress biomarkers is cortisol, a glucocorticoid hormone released by the adrenal glands that is central to the physiological stress response. Free cortisol can be measured in saliva and has been the biomarker of choice in stress studies measuring the function of the HPA axis. Humans generally release cortisol in a tightly-regulated pattern that follows a diurnal rhythm controlled by the HPA axis. In a normal diurnal rhythm, cortisol rises sharply upon awakening, peaks within about 30 minutes of arousal, and slowly falls throughout the day [8]. The spike in cortisol concentration in the morning is referred to as the cortisol awakening response (CAR). While acute physical and psychological stressors can cause fluctuations in this pattern, chronic psychosocial stress can lead to long-term dysregulation of cortisol, including elevation or depression of the CAR [9]. Long-term cortisol dysregulation is associated with negative health consequences, such as increased visceral adiposity and obesity, increased blood glucose, mood disorders and higher risk of cardiovascular disease [10,11]. Despite the widespread focus on cortisol, there is still debate among researchers about procedures for collecting saliva samples. Today, most protocols involve collection of three or four daily samples over the course of at least two consecutive days in order to account for daily diurnal fluctuations and intra-individual variability, and to allow for measurement of wake-up levels, CAR and total daily cortisol concentration [8].

Despite the growing use of biomarkers to measure chronic psychosocial stress in a variety of settings, little is known about links among identified psychosocial stressors, stress buffers (for example, family support), and elevated stress biomarkers among immigrant and minority populations in the United States. Given the role that stress plays in the development and progression of disease [12-14], and the known health disparities in immigrant and minority populations in the United States compared to the white population [15-18], biomarkers are a useful tool for measuring health status related to psychosocial stress at population and individual levels. It is unfortunate that relatively few studies have focused on minority and immigrant populations because the incorporation of biomarkers could aid our understanding of factors that contribute to health disparities.

### Latino immigrants and psychosocial stressors in Oregon

Despite the size of the Latino population in the United States (at 15% of the U.S. population; [19]), behavioral and physical health data remain sparse for this group. This is especially true for the 37% of Latinos in the United States who are foreign born [20]. In Oregon, where the present study was conducted, Latinos are also the largest ethnic/racial minority group (at 12%; [20]) although, like 21 other states that are recent sites of rapid immigrant population growth, Oregon has limited experience with large influxes of immigrant newcomers. Existing racial and ethnic health disparities in Oregon and elsewhere are a testament to the challenges that both newly arrived and established minority groups face in accessing health care and prevention services [18]. These disparities are partially a result of barriers to and lack of available care, and are also related to profound stressors commonly experienced by immigrant and minority families.

Many immigrant families report experiencing a range of psychosocial stressors related to low SES, limited economic mobility, discrimination, challenges of adapting to life in the United States (acculturation), employment uncertainty, worry about families in countries of origin and concerns about legal status. In states like Oregon, there may be few formal buffers (for example, culturally and linguistically competent services, established immigrant enclaves) against such stressors.

Our investigation of stressors and stress is informed by McEwen’s concept of allostatic load or the “price the body pays over long periods of time for adapting to challenges” [21]. This approach distinguishes between “stressors” (the circumstances to which individuals are exposed) and “stress” (the extent to which an individual is challenged to maintain function). The present study pays particular attention to stressors related to acculturation, discrimination, SES, lifestyle incongruity and social support in relation to effects for Latino immigrant adults’ stress biomarkers and anthropometric measures, some of which also are recognized health outcomes (for example, measures of central and total body adiposity); key stressors considered in the present research are briefly described below.

*Acculturation* is a multidimensional construct that describes phenomena resulting from continuous contact between groups of individuals from different cultures, including subsequent changes in the cultural patterns of one or both groups [22]. Acculturation is multifaceted and includes such factors as linguistic proficiency, language use, nativity and culture-related behavioral preferences, among others. Importantly, acculturation serves as a marker for other psychosocial processes (for example, ethnic identity processes, experiences with structural barriers such as poverty, and difficulty obtaining work authorization) that may link acculturative stressors with biological stress and health outcomes [23].

Numerous studies have illustrated the ways in which *discrimination* and *stigma* - social processes linked to the reproduction of inequality and exclusion - are an intrinsic part of life in the United States and affect minority groups on a daily basis [17,24]. Discrimination may exert stressful effects directly upon individuals through actual or anticipated discriminatory social interactions, and indirectly through structuring SES and other forms of opportunity [25]. Low SES, in turn, has been associated with chronic activation of the stress-response, greater health risks and poorer health across the lifespan [26]. The negative mental health effects of prejudice and stigma on adults and adolescents are well-documented, with links between perceived discrimination and poor physical health investigated most often among African-Americans, though findings remain mixed [18]. Importantly, evidence indicates that discrimination stress has negative health effects above and beyond that of perceived stress related to unpredictability and lack of control [27].

*Lifestyle incongruity* (LI) reflects attempts by individuals to maintain a lifestyle inconsistent with their economic standing [3,28]. Originally studied in populations experiencing rapid culture change, a growing body of research has documented stress and negative health effects associated with the displacement of traditional markers of status and prestige by novel ones, such as consumer and luxury goods [3,28-30]. Several studies have identified significant deleterious effects of high LI on blood pressure [31-35] and immune function [3,30]. The majority of these studies were conducted with populations outside the United States undergoing rapid economic development [3,30,32,33,35]. Few if any studies have been conducted among immigrants in the United States.

*Social support* has been recognized as an important mediator of stress on health. Research indicates that increases in social affiliation with supportive others can substantially reduce stress and its negative health effects. Extensive research has documented that a dearth of support, in contrast, may lead to chronic activation of the stress response, leading to immunosuppression and greater risk of disease, as well as to an overly active sympathetic nervous system leading to higher blood pressure [36]. Cross-cultural prospective studies confirm that the impact of social isolation on life expectancy appears to be as large as cigarette smoking, hypertension, obesity and lack of physical activity. *Social isolation* is generally less prevalent in non-Western and non-industrialized societies. Once in the United States, however, immigrants whose social ties have been attenuated through the process of immigration may have smaller social networks than they are accustomed to and experience higher psychosocial stress [37], though little is presently known about the effects on immigrants’ stress and health.

## Hypotheses

The majority of the literature to date on psychosocial stress and cortisol has not been conducted with Latino immigrant populations in the United States. Our hypotheses below extrapolate from existing cortisol literature, as well as draw on the few studies that have been conducted among Latino youth [38]. Literature on acculturation and health among Mexican immigrants has shown that more time in residence leads in the United States to increased risk of negative health outcomes [23,39]. Our hypotheses also reflect studies [39] that have relied on other biomeasures (for example, blood pressure, cholesterol, waist circumference (WC), body mass index (BMI), glucose and glycosylated hemoglobin) and used these to hypothesize relationships among Latino immigrants’ cortisol, acculturation, and health. Our hypotheses are as follows:

1. Dysregulated cortisol (blunted CAR as measured by lowered AUCg) will be significantly correlated with psychosocial stressors (related to discrimination, poverty, SES and/or higher lifestyle incongruity).

2. More time in residence and greater English language engagement will significantly and positively correlate with dysregulated cortisol, measured by AUCg.

3. As elevated cortisol has been shown to relate to the body composition measures most often implicated in increased cardiovascular disease risk (that is, greater central adiposity; [40]), elevated daily cortisol production (AUCg) among study participants will positively and significantly relate to measures of central fat (WC and waist-stature ratio (WSR)) and overall fat (body mass index; BMI) among both men and women.

## Methods

### Study population

As mentioned, Latinos are the largest minority group in Oregon and the population is rapidly increasing [19]. According to available data, between 85% and 95% of Latinos in Oregon are of Mexican origin [19,41]. Oregon’s Latino immigrant population also includes a growing number of indigenous Mexicans [42]. In the current study, 96% of participants reported being of Mexican origin and 15% (n = 18) reported being of indigenous ancestry (for example, Mixtec, Zapotec, Purepecha). (See Table 1 for sociodemographic characteristics of study participants.)

**Table 1:** **Descriptive statistics for anthropometric, health and lifestyle data among participantsparticipants**^**a**^

**Characteristic**	**Males (n = 23)**	**Females (n = 38)**
Age (*years*)	37.17 (12.8)	33.84 (10.5)
Time in residence (*years*)	12.48 (10.1)**	6.97 (5.5)
Age at arrival in U.S. (*years*)	23.77 (10.6)	25.38 (12.7)
Height (*cm*)	164.67 (6.5)***	155.95 (7.4)
Weight (*kg*)	77.96 (15.0)*	70.3 (13.3)
BMI (kg/m^2^)	28.65 (4.5)	28.99 (5.5)
Waist circumference (WC; *cm*)	95.28 (13.8)	89.58 (14.0)
Waist-to-stature ratio (WSR)	0.58 (0.08)	0.58 (0.10)
Glucose (*mg/dL*)	76.61 (9.5)	80.55 (14.2)
Total cholesterol (*mg/dL*)	133.61 (18.9)	144.54 (25.8)
Systolic blood pressure (*mm Hg*)^a^	115.91 (12.1)	110.23 (17.4)
Diastolic blood pressure (*mm Hg*)^a^	72.71 (8.7)	72.57 (9.8)
Cortisol AUCg1	1.39 (1.36)^b^	1.71 (1.3)
Cortisol AUCg2	1.36 (0.99)^b^	1.48 (1.4)
Cortisol AUCg average	1.38 (0.80)^b^	1.60 (1.1)

^a^All values presented as mean (SD) unless otherwise noted.

^b^Mean (SD) only includes subset of cases where cortisol samples were provided.

Differences between males and females are statistically significant at: * *P*<0.05; ** *P*<0.01; *** *P*<0.001.

### Participants

This community-based participatory research project involved Latino immigrant farmworkers and took place in two phases in the northern Willamette River Valley in Oregon. The first phase was conducted at two study sites: a small rural community (pop. 8,200) and on the outskirts of one of Oregon’s medium-sized cities (pop. 149,000). The second phase took place at a third study site within an established Latino ethnic enclave (pop. 22,000). In total, 119 adult volunteers participated in the study (43 men and 76 women). To be eligible, participants had to be 18 years of age or older and, if female, not pregnant. The study involved collaboration with a well-respected local farmworker housing organization.

### Recruitment and assessment

The study sample was drawn from a non-probability design and recruitment was conducted through trusted social networks, and by recruiters who are the staff of our partner organization and share characteristics of the target study population [43-45]. On a single day, residents participated in a health assessment and responded to a 20-minute interview. Senior staff from the Oregon Social Learning Center (OSLC) Latino Research Team (LRT) and the farmworker organization collaborated on the design of the interview. Due to its brevity, specific questions were drawn from a larger assessment battery that had been extensively developed by the LRT for use with the Latino population in Oregon [46]. A focus group composed of Latino immigrant farmworkers reviewed the questionnaire, and changes were made per focus group findings.

The Institutional Review Boards at OSLC and the University of Oregon approved all research protocols and all participants provided written consent prior to the assessment. All respondents were assessed in Spanish.

The health exam involved measures of blood pressure, height, weight and WC. Blood pressure was measured using an Omron HEM-422 CRLC manual inflation oscillometric blood pressure monitor (Vernon Hills, IL, USA) using established procedures. Blood pressure was measured two times per participant. Stature, weight and WC were measured using standard procedures [47]. BMI was calculated as mass divided by height in meters squared (kg/m^2^) and WSR was calculated from waist circumference and height. Staff used a disposable lancet to prick the participant’s finger to collect a drop of blood for the immediate measurement of fasting glucose and total cholesterol (all participants had fasted > 8 h). Each participant met with a health educator to review their health values. Participants with measures indicating need for follow-up were referred to partnering public health agencies.

Assessors then provided a one-on-one saliva collection demonstration. Participants were instructed in the steps to collect their own saliva samples by passive drool in 1.7 mL Eppendorf tubes. Participants collected samples three times per day over two consecutive days, with collections immediately upon awakening, 30 minutes after waking, and in the evening prior to going to sleep. Participants were instructed to record exact collection times, and were requested not to eat, smoke, drink or engage in physically-exhausting activity within 30 minutes of sample collection, as these activities can influence cortisol concentration [8]. This sampling protocol has been shown to adequately capture the cortisol awakening response [48]. The participant response rate was 75%.

Project staff made reminder calls to participants the evening before and the day of each saliva collection. After collection, all samples were stored in participants’ home refrigerators for no more than one week until transported to the University of Oregon, where samples were stored in a −30°C freezer until analyzed. Preservative-free salivary cortisol has been shown to be stable at room temperature for up to four weeks [40]. All cortisol samples were analyzed with a salivary cortisol ELISA kit (Salimetrics, State College, PA, USA) in the Snodgrass Laboratory at the University of Oregon.

The interview contained self-report sociodemographic, social support, perceived discrimination and acculturation items. *Discrimination Stress* was measured through a single item asking whether respondents had been treated as if inferior in the last three months and, if so, the degree of stress they experienced in relation to the event(s) (1 = no stress to 5 = extreme stress). *Family Support\* was evaluated through a single item asking whether they can count on family if there are problems (1 = completely agree to 5 = completely disagree). *SES* was computed through a z-scored variable combining household income and education; occupation was excluded as there was little variance among the farm worker sample. A factor score reflecting *English Language Engagement* combined items regarding the extent to which participants speak, read and write English and engage in English-language activities (1 = not at all to 5 = very well/a lot). *Time in Residence* in the United States (TR) and *Age upon Arrival* in the United States were computed from self-reported years and months. Participants who remit or send money to home countries were asked whether *Remitting Money* caused them *stress* (1 = never to 5 = always). Finally, a measure of *lifestyle incongruity* was derived from participants’ responses to two sets of questions: 1) participant self-rated importance (1 = not important to 3 = very important) of owning/having each of 18 material objects (for example, stereo, car, driver’s license) to living a good life in the United States; and 2) how many of each item they possessed. A final variable was computed reflecting the difference between the two measures. Though previous researchers included an evaluation of economic standing in their LI analyses, research with recent Latino immigrants in the United States has identified that financial strain related to an inability to meet one’s own aspirations correlates more strongly with stress and negative outcomes than do measures of SES [49]. For this reason, we slightly adapted the LI variable to better assess the “fit” between an individual’s perception and realization of material success in the United States, a central component of the “American Dream” often ascribed to by newly arrived immigrants in the United States [50].

### Statistical analyses

This study investigated relationships between psychosocial stressors (for example, perceived discrimination) and salivary cortisol area under the curve with respect to the ground (AUCg). Saliva samples were available from a participant sub-sample composed of 61 adult (18 to 69 years of age) participants (23 male and 38 female), and analyses focused on 58 Latino immigrants (22 men and 36 women), after excluding three U.S.-born participants. For cortisol, one outlier individual cortisol sample concentration (that is, >2.5 SD) was replaced with a less extreme cortisol value (that is, next highest cortisol value in the sample) to normalize the distribution. As age, WC and other body composition measures can co-vary with cortisol, Pearson’s correlations were run with each cortisol sample concentration with respect to recorded body composition values. The data were analyzed separately by sex.

AUCg was measured following the procedure of [51]):

(1) $$\text{AUCg}\;\text{=}\;\left(\left(\left({\text{m}}_{1}\;\text{+}\;{\text{m}}_{2}\right)\;\text{x}\;{\text{t}}_{1}\right)/2\right)\;\text{+}\left(\left(\left({\text{m}}_{2}\;\text{+}\;{\text{m}}_{3}\right)\;\text{x}\;{\text{t}}_{2}\right)/2\right)$$

where m = measured cortisol concentration and t = time between samples

AUCg variables were calculated for Day 1 (AUCg1) and Day 2 (AUCg2) and an average for the two days, and then run as Pearson’s correlations in the same manner as individual samples. After determining that WC was the strongest confounder for women and age for men, Pearson's partial correlations were used for determining relationships between AUCg variables and independent variables. AUCg has been shown to be an accurate measure of the entire day’s free cortisol levels [51]. Data were analyzed using SPSS version 17.0 (IBM Corporation, Armonk, NY, USA).

## Results

Descriptive statistics for anthropometric, cortisol and sociodemographic data for men and women are presented in Table 1. Mean AvgAUCg levels for men were 1.38 mg/L and women’s mean AvgAUCg values were 1.60 mg/L. Intra-individual variability was low, with Day 1 and two samples highly correlated (*P*<0.01). Days 1 and 2 AUCg measures were highly positively correlated for men (*P*<0.01) and showed a trend for women (*P* = 0.075) (Tables 2 and 3). Men’s and women’s mean cortisol AUCg concentrations showed a trend for differences (*P* = 0.089), women’s being slightly higher. The study population did not exhibit values out of the normal range for adults of comparable age in the United States based on manufacturer assay kit information (Salimetrics, State College, PA, USA). Cortisol AUCg concentrations from both days were unrelated to smoking, drinking alcohol or oral contraceptive use.

**Table 2:** **Correlation matrix for anthropometric and lifestyle variables for females**^**a,b**^

	**AUCg1**	**AUCg2**	**AvgAUCg**	**Height *cm***	**Weight *kg***	**WC *cm***	**BMI *kg/m***^***2***^	**WSR**	**TR *years***	**Age *years***	**LI**
AUCg1	1	0.292	0.861***	0.090	−0.374^*^	−0.442**	−0.406*	−0.410*	−0.049	−0.038	0.322
AUCg2		1	0.825***	−0.192	−0.10	−0.083	0.007	−0.018	0.109	0.118	0.409*
AvgAUCg			1	−0.072	−0.288	−0.315*	−0.236	−0.254	0.043	0.055	0.473*
Height (*cm*)				1	0.186	−0.298	−0.286	−0.533***	0.054	−0.219	−0.369
Weight (*kg*)					1	0.774***	0.885***	0.628***	0.313	0.265	−0.013
WC (*cm*)						1	0.898***	0.965***	0.193	0.510***	−0.034
BMI (*kg/m*^*2*^)							1	0.868***	0.181	0.364*	0.180
WSR								1	0.145	0.513***	0.106
TR (*years*)									1	0.181	0.252
Age (years)										1	0.075
LI											1

^a^Abbreviations: *BMI*, body mass index; *LI*, lifestyle incongruity; *TR*, time in residence; *WC*, waist circumference; *WSR*, waist-to-stature ratio ^b^Correlations are statistically significant at: ^*^P<0.05; ^**^P<0.01; ^***^P<0.001.

**Table 3:** **Correlation matrix for anthropometric and lifestyle variables for males**^**a,b**^

	**AUCg1**	**AUCg2**	**AvgAUCg**	**Height *cm***	**Weight *kg***	**WC *cm***	**BMI *kg/m***^***2***^	**vWSR**	**TR *years***	**Age *years***	**LI**
AUCg1	1	0.571**	0.861***	.182	−0.018	−0.167	−0.109	−0.237	0.237	−0.440*	0.063
AUCg2		1	0.909***	−0.033	−0.195	−0.134	−0.237	−0.145	−0.018	−0.343	−0.272
AvgAUCg			1	0.071	−0.130	−0.168	−0.202	−0.210	0.110	−0.436*	−0.139
Height (*cm*)				1	0.559**	0.431*	0.1270	0.145	−0.323	−0.329	0.062
Weight (*kg*)					1	0.870***	0.888***	0.767***	0.073	0.121	−0.012
WC (*cm*)						1	0.813***	0.954***	0.246	0.147	−0.047
BMI (*kg/m*^*2*^)							1	0.849***	0.350	0.283	−0.061
WSR								1	0.279	0.387	−0.078
TR (*years*)									1	0.394	0.321
Age (years)										1	0.521*
LI											1

^a^Abbreviations: *BMI*, body mass index; *LI*, lifestyle incongruity; *TR*, time in residence; *WC*, waist circumference; *WSR*, waist-to-stature ratio;

^b^Correlations are statistically significant at: ^*^P<0.05; ^**^P<0.01; ^***^P<0.001.

For women, several anthropometric measures were significantly associated with AUCg1 (Table 2). AUCg1 was negatively correlated with both WC (*P*<0.01) and WSR (*P*<0.05). AUCg1 was also negatively correlated with weight (*P*<0.05) and BMI (*P*<0.05). In subsequent analyses, for females, WC was controlled for as it was the strongest confounder.

For men, age, but not anthropometric variables, was significantly (and negatively) associated with AUCg1 and AvgAUCg (Table 3). Age was controlled for in further analyses with independent variables.

**Figure 1:**
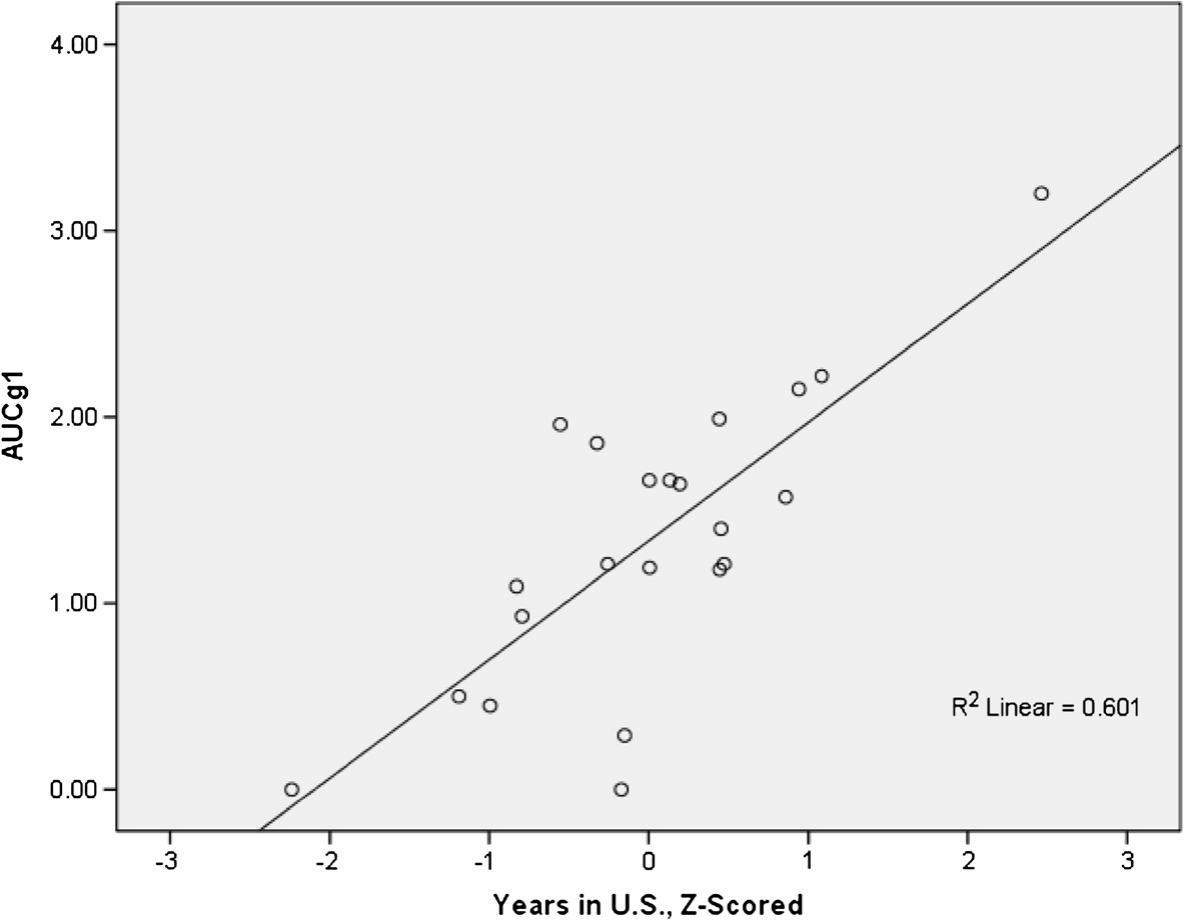
**Men’s cortisol AUCg1 with respect to years spent in the U.S., adjusted for age.** Several other independent variables showed associations with AUCg variables when adjusted for WC using partial correlations. For women, the greater the lifestyle incongruity, the greater the area under the curve for Day 1 (*P* = 0.053*, r*^2^ = 0.409); there was no relationship between AUCg1 and lifestyle incongruity in men (*P* = 0.235, *r*^2^ = −0.241). For all measures of AUCg, there were no significant associations with English language engagement, social support, SES, family support, discrimination or remittance stress in either women or men.

Time spent in the United States was a positive predictor of AUCg1 (*P*<0.01) (Figure 1) in men only, independent of age at sample. Men who immigrated at a younger age, regardless of how long they had lived in the United States, had lower AUCg1 (*P*<0.05).

## Discussion

Results provided mixed support for our hypotheses. Interestingly, cortisol AUCg data did not correlate with body composition measures as expected. Several studies have shown that increased cortisol concentrations are correlated with increased visceral adiposity, overweight and obesity [52]. In the present study, however, AUCg was highly negatively correlated with WC in women, as well as WSR, BMI and weight. Actually, this is unsurprising, given previous evidence that dysregulation in HPA function leads to increasing visceral adiposity [1]. Most studies of HPA dysregulation have focused on elevated cortisol levels, yet cortisol dysregulation can take many forms, including low overall daily cortisol concentrations. However, our findings showed that men had no significant associations between cortisol measures and body composition. It is possible that nutrition and exercise variables that were not measured in the current study account for the lack of significant relationships among men between body composition measures and cortisol, and for women’s higher WC and BMI. There may also be other mediators (such as social support) that influence relationships among stress, cortisol and body composition measures that were not captured in the present study.

The finding that men who have been in the United States the longest have the most blunted cortisol curves is striking because it supports other studies showing that more time in residence is linked with negative health outcomes, such as higher depression and substance abuse [53,54], as well as high blood pressure, and higher cholesterol as a result of increased allostatic load over time [39]. It is important to note that the amount of time in residence was independent of the age at arrival in this population, meaning that older men have not necessarily been in the United States the longest and younger men did not necessarily arrive more recently. Arriving at an older age or spending more time in the United States may result in men being more susceptible and exposed to acculturative stressors. It is also possible that older men are also dealing with additional stress involved with trying to earn enough to bring the rest of their families with them, since men tend to be the first members of families to immigrate to the United States, and it is likely that older men have larger families at the time of immigration.

The bulk of research shows that arrival in the United States at older ages is protective against poor outcomes often associated with greater acculturation to life in the United States (for example, greater risk of substance use, unsafe sexual behavior, depression; [55]). However, this study raises the possibility that arrival at an older age carries with it unique vulnerabilities. It is possible that there is a developmental window during which time an individual’s biological response to stress is relatively plastic but becomes less so with age [56]. For younger men with more plasticity in their stress response, they may be more able to adapt to a life-changing event, such as immigration and subsequent settlement, without inducing a dysregulated cortisol pattern. This finding also may be unique to contexts such as exist in Oregon, which has few established Latino immigrant communities and institutional supports for the successful integration of newly arrived Latino immigrants, such as exist in states like California, Texas and Florida [57]. In this context, the presence of greater environmental stressors (for example, discrimination, language barriers, [45]) may exact an even greater toll on older immigrants whose age in home countries or established immigrant enclaves in the U.S., might otherwise confer authority or social status.

It is noteworthy that women who reported the greatest disparities between items they owned versus items they believed important to living a good life in the United States were shown to have a lower AUCg. Previous research with a Latino population showed that chronic psychosocial stress led to diminished CAR [58,59], and it may be that stress related to LI contributed to a blunted cortisol response. In the present study it is unclear why this pattern was identified among woman and not men, though a potential explanation may be found in other studies identifying links between high LI and elevated blood pressure related to women’s low expression of “negative” emotions, including anger suppression [32,60]. Future studies are needed to determine if women’s chronic psychosocial stress related to anger suppression (or other forms of emotional expression) in the face of high lifestyle incongruity may account for women’s blunted cortisol as well as for elevated blood pressure [61,62].

Findings from the current study raise important questions about the gendered nature of stress pathways, and add to a small but growing literature on psychosocial stressors and physiological responsiveness among Latino immigrants. Our work to date indicates several distinct pathways for Latino immigrant women and men, with discrimination stress predicting elevations in Epstein-Barr virus antibodies (an immunological measure) and systolic blood pressure among men [63] and relating to increased glucose and BMI among women [64]. Findings in relation to C-reactive protein (a measure of inflammation) show that greater hours of reported daily TV viewing significantly predicted elevated CRP levels (after controlling for WSR) among men but not women [65]. Several potential explanations exist for these gender differences. It may be that women have different outlets for stress responses than men, such as depression or changes in eating behavior [27], or that women’s biology moderates stress responses differently than men’s (an idea suggested by several studies; [14,66]). Clearly, much remains to be explored in relation to environmental stressors related to discrimination and acculturative processes, Latino women and men’s health measures, and potential mediators, such as depression, anger suppression, or nutrition and activity levels.

One interesting finding was that certain measures were significantly related to only one day’s area under the curve or the average, but not all, which is particularly interesting given that all days’ cortisol measures were significantly correlated to one another. A possible explanation for this lies in a dissection of the day’s cortisol output. The CAR is a very tightly regulated process, dictated strongly by genetics. It is very difficult to change the CAR absent chronic stressors and/or depression. In our study, the CAR was captured by the first morning and 30-minute post wakeup measures. Evening cortisol levels are more easily affected by day-to-day stressors, and thus most likely to differ on a daily basis [67]. Given that cortisol concentrations are at their lowest in the evenings, day-to-day evening changes that are small in magnitude were not enough to cause large enough overall changes in AUC between days, but were enough to change how daily cortisol AUC values related individually to other variables and might explain why some variables were associated with one day of AUC, but not another even when AUC day one and two are highly correlated.

This study has several important limitations. First, the sample size is small and non-representative, which limits the generalizability of the findings. Second, the current study collected three saliva samples over two consecutive days, but may have benefitted from additional samples over more days. Third, the assessment instrument for the current study was collaboratively designed with community partners with the goal of brevity and minimal participant burden. However, as a result, current analyses relied on a single item for several measures (for example, for family support, discrimination), which, in some instances, may have consequences for results. In addition, because of the length of the assessment, key variables of interest, such as food frequency, exercise, negative mood states (for example, depression, anger) and sleep, were not appraised. Future studies of links among cortisol, psychosocial stress and health among Latino immigrants would do well to more fully assess these factors.

Although preliminary, results from the present study suggest that chronic psychosocial stress plays an important role in structuring health risk among Latino immigrants in Oregon. However, further studies are required to determine the long-term health consequences of dysregulated cortisol rhythms in this population. Ultimately, this research is intended to contribute to knowledge about pathways linking stress to poor health outcomes. Our focus on an immigrant Latino sample is designed to give us insights into unique areas of vulnerability and resilience in Latino women and men in regards to stress. Through identifying patterns that may be specific to sub-groups, such as Latino immigrants, we can inform public health practice and policy to better protect against potential stress-related negative health outcomes, though much work remains to better identify those links.

## Abbreviations

AUC: Area under the curve; AUCg: Area under the curve with respect to the ground; AUCg1: AUCg day 1; AUCg2: AUCg day 2; AvgAUC: Average of day 1 and 2 AUCg; BMI: Body mass index; CAR: Cortisol awakening response; ELISA: Enzyme-linked immunosorbent assay; HPA: Hypothalamic-pituitary-adrenal; LI: Lifestyle incongruity; LRT: Latino Research Team; OSLC: Oregon Social Learning Center; SES: Socioeconomic status; SNS: Sympathetic nervous system; TR: Time in residence; WC: Waist circumference; WSR: Waist-stature ratio.

## Competing interests

The authors declare that they have no competing interests.

## Authors’ contributions

ECS, HHM, RAJ, and JJS were responsible for study concept and design. HHM, CRM, JME, and JJS obtained funding. ECS, HHM, LEI, and JJS acquired data. ECS performed laboratory analyses, and ECS and HHM performed statistical analyses. ECS, HHM, CRM, JME, and JJS were involved with interpretation of data. ECS, HHM, and JJS drafted the manuscript. CRM, JME, and RAJ provided critical revisions to the manuscript for intellectual content. Administrative supervision was provided by CRM, RAJ, and JJS. All authors read and approved the final manuscript.
